# Time to listen: harnessing podcasts for stakeholder engagement in circadian and sleep science

**DOI:** 10.1038/s44323-026-00077-1

**Published:** 2026-06-03

**Authors:** Carolina Guidolin, Manuel Spitschan

**Affiliations:** 1 TUM School of Medicine and Health, Department Health and Sports Sciences, Chronobiology & Health, Technical University of Munich, Munich, Germany; 2 Max Planck Institute for Biological Cybernetics, Max Planck Research Group Translational Sensory & Circadian Neuroscience, Tübingen, Germany; 3 TUM Institute for Advanced Study (TUM-IAS), Technical University of Munich, Garching, Germany; 4 TUMCREATE Ltd., Singapore, Singapore

**Keywords:** Psychology, Psychology, Science, technology and society

## Abstract

Circadian and sleep research spans molecular to applied domains, yet communication gaps persist across disciplines and with the public. This article argues that podcasting is a versatile tool for bridging these gaps. We outline how podcasts can disseminate emerging research, communicate established evidence and uncertainty, support university teaching, and promote behaviour change. We also discuss challenges, including quality control, reach, sustainability, and funding, situating podcasting within contemporary science communication practice.

## Introduction

Over the past two decades, podcasts have emerged as a popular medium for communicating scientific ideas and findings^1^. Put simply, a podcast is a digital audio recording that can be listened to on a computer or portable device, such as a smartphone. Podcasts are typically distributed using really simple syndication (RSS) feeds, which allow listeners to subscribe and receive automatic updates when new episodes become available^2^. Several factors explain the increasing popularity of podcasts as a science communication tool. Contrary to radio broadcasting or TV media, podcasts are available “on demand” and can be enjoyed anytime and anywhere, making them well-suited to varied schedules. Most podcasts are freely accessible online, enabling a global reach across diverse age groups and geographical regions. Their format is also highly adaptable, ranging from monologues and expert interviews to personal storytelling combined with scientific discussion, which allows the science communicator (i.e., the podcast host) to tailor content to niche audiences and convey more of their personality than is typically possible in written formats. Lastly, compared to television or print media, science podcasts often encourage listeners to comment or provide feedback on their episodes, thus promoting an active audience community and creating a personal relationship between the science communicator and the listener^1^. These advantages have led to both individual scientists launching independent podcasts, institutions incorporating podcasting into their communication strategies, and a variety of peer-reviewed journals adopting this tool to highlight and discuss published findings, such as *Nature*, *The Lancet*, and *Science*^3,4^.

Circadian and sleep science provides a timely case study for this medium. Since the late 1990s and early 2000s, landmark discoveries such as the characterisation of clock genes and the identification of melanopsin-containing intrinsically photosensitive retinal ganglion cells have transformed our understanding of the mechanisms underlying circadian timekeeping and the influence of light exposure on sleep. These advances, and the research they inspired, have had far-reaching implications across diverse fields: clinicians considering the optimal timing of medication, lighting designers and architects developing circadian-friendly illumination systems, ecologists examining animal behaviour in light-polluted environments, and agricultural scientists optimising crop performance, among others.

Beyond academia, circadian science has entered public discourse. Major news outlets have reported on its relevance to health and well-being^5–7^; social media trends, such as “sleep syncing,” have gained viral traction; and popular science books on the subject have reached broad audiences. For example, *Life Time* by Russell Foster featured on the *Sunday Times* bestseller list, while the recent book *The Inner Clock* by Lynne Peeples has received extensive coverage across popular media^8,9^. Meanwhile, the pace of discovery in circadian research is accelerating, with advances emerging simultaneously across molecular, clinical, ecological, and applied domains. Yet, substantial knowledge gaps persist, underscoring the need for effective communication with stakeholders within and beyond academia. Despite the growing societal relevance of circadian and sleep science, the field still struggles to convey its insights across disciplines and to the public.

**Fig. 1:**
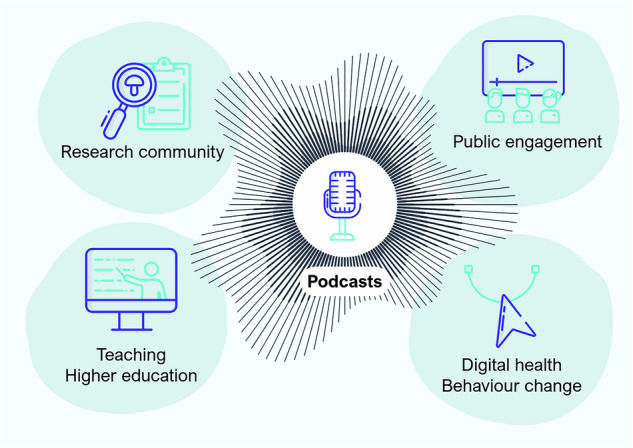
Podcasting as a communication bridge.

In this article, we explore the potential for podcasting as a tool to (1) share emerging research with fellow scientists; (2) communicate established findings and uncertainties to the public; (3) support the incorporation of circadian and sleep science into university curricula; and (4) promote behaviour change as part of digital behavioural interventions (Fig. 1). We also highlight potential pitfalls of podcasting, and compare and highlight existing examples of podcasts in the field (Table 1). Finally, we highlight our own podcast, *Light O'Clock* (Table 2, Box 1).

**Table 1:** Overview of currently active chronobiology podcasts in the English language (January 2026)

Name	Host	Episode length	Format	Website
Light O’Clock	Carolina Guidolin	Ca. 30 minutes	Interviews with scientists and storytelling	https://tscnlab.org/podcast
The SRS podcast	Jesse Cook	Ca. 60 minutes	Discussion of publications by authors	https://sleepresearchsociety.org/career-advancement/srs-podcasts/
CHRONO: MEDICINE (formerly 247Muscle)	Jan-Frieder Harmsen	Ca. 30–90 minutes	Interviews with scientists	https://open.spotify.com/show/1u5Sh1KNFvpmySsf7BstcZ
Performance around the clock	Satchin Panda	Ca. 60–120 minutes	Interviews with scientists	https://open.spotify.com/show/2GXeqcvxrn3TJYI8P3Va7H
Talking sleep	Seema Khosla	Ca. 30–45 minutes	Interviews with scientists, clinicians and healthcare professionals	https://aasm.org/professional-development/talking-sleep-podcast/
The Matt Walker podcast	Matt Walker	Ca. 30–90 minutes	Mixed format, including interviews with guests	https://themattwalkerpodcast.buzzsprout.com/1821163/episodes
Sleep science podcast	Penny Lewis	Ca. 30–50 minutes	Interviews with scientists	https://sleepsciencepodcast.buzzsprout.com/787211/episodes
Sleep is a skill	Mollie McGlocklin, Mollie Eastman	Ca. 45–60 minutes	Interviews with industry leaders	https://podcasts.apple.com/us/podcast/the-sleep-is-a-skill-podcast/id1501931597
Sleep Science Today	Andrew Colsky	Ca. 45 minutes	Interviews with sleep scientists, doctors, and wellness experts	https://nationalsleepcenter.com/podcast/

The listed podcasts were identified using a structured but non-exhaustive search of the podcast search engine *Listen Notes*. Searches were conducted using the terms “sleep science” and “circadian science,” excluding podcasts with fewer than five episodes. For “sleep science,” only the top 20 search results were reviewed, and podcasts primarily designed as sleep aids were excluded.

**Table 2:** Episodes in the Light O’Clock podcast (October 2025)

Guest	Title	DOI
Manuel Spitschan	Light O'Clock (Season 1) – Episode 1: Light exposure – why should we care?	10.17617/1.n378-5b97
Maydel Fernandez-Alonso	Light O'Clock (Season 1) – Episode 2: Light’s journey through the eye	10.17617/1.16tx-3391
Robert Lucas	Light O'Clock (Season 1) – Episode 3: Using light to tell time of day	10.17617/1.c9aw-4p24
Elise McGlashan	Light O'Clock (Season 1) – Episode 4: Melatonin – the night time hormone	10.17617/1.0ctp-rq05
Rafael Lazar	Light O'Clock (Season 1) – Episode 5: Shedding light on... pupil size across the lifespan	10.17617/1.c778-zm67
Manuel Spitschan	Light O'Clock (Season 1) – Episode 6: You ask, we answer – special Q&A episode	10.17617/1.x0h5-gg57
Orie Shafer	Light O'Clock (Season 2) – Episode 1: Cave studies and fruit flies – the history of chronobiology	10.17617/1.e338-v573
Renske Lok	Light O'Clock (Season 2) – Episode 2: Sleep and circadian rhythms – same or different?	10.17617/1.awh0-hz11
Anna M. Biller	Light O'Clock (Season 2) – Episode 3: Early birds and night owls – what is chronotype?	10.17617/1.mc7y-4k26
Laura Kervezee	Light O'Clock (Season 2) – Episode 4: In and out of sync	10.17617/1.cjvv-fc13
Daniel Smith	Light O'Clock (Season 2) – Episode 5: Shedding light on... circadian rhythms and mental health	10.17617/1.t9ph-t492
Till Roenneberg, Sara Montagnese & Andrew Coogan	Light O'Clock (Season 3) – Episode 1: Tick-tock trouble – How clock changes affect our health	10.17617/ebny-b731
Luísa Klaus Pilz	Light O'Clock (Season 3) – Episode 2: City lights, country nights – Circadian rhythms in urban and rural life	10.17617/4awr-1768
Ray Najjar	Light O'Clock (Season 3) – Episode 3: Blurred vision – The myopia epidemic and indoor lifestyles	10.17617/b98p-9565
Anna M. Biller	Light O'Clock (Season 3) – Episode 4: Too early to think? Why school start times matter for sleep and learning	10.17617/0jy1-ye56
Travis Longcore, Barbara Harding & Karolina Zielinska-Dabkowska	Light O'Clock (Season 3) – Episode 5: Drowning in light – The hidden costs of light pollution	10.17617/hpwg-t981
Anna Wirz-Justice	Light O’Clock (Spotlight) – The dark side of daylight (Daylight Awareness Week 2024)	10.17617/1.pm90-9074
Amy Ferguson, Patricia Pelosi & Nomhle Nhlapho	Light O'Clock (Spotlight) – Research, rooted in reality – The power of lived experience in scientific studies	10.17617/qkpb-z786
Marie-Luise Schreiter (co-host)	Light O'Clock (Live episode) – Night owl or early riser? A live podcast episode	10.17617/1.fpqe-fr67
Delainey Wescott, Hester Parr & Hayden Lorimer	Light O'Clock (Spotlight) – Winter blues: SAD and how to winter well	10.17617/1.78xg-d729
Anna M. Biller & Priji Balakrishnan (co-hosts)	Light O'Clock (Interacting with Daylight Mini-Series) – Episode 1: Geolocation	10.17617/1.5q1h-ad89
Anna M. Biller & Priji Balakrishnan (co-hosts)	Light O'Clock (Interacting with Daylight Mini-Series) – Episode 2: Culture & behaviours	10.17617/1.jman-gd65

Box 1 *Light O’Clock* – a podcast focusing on the circadian clock and the influence of light

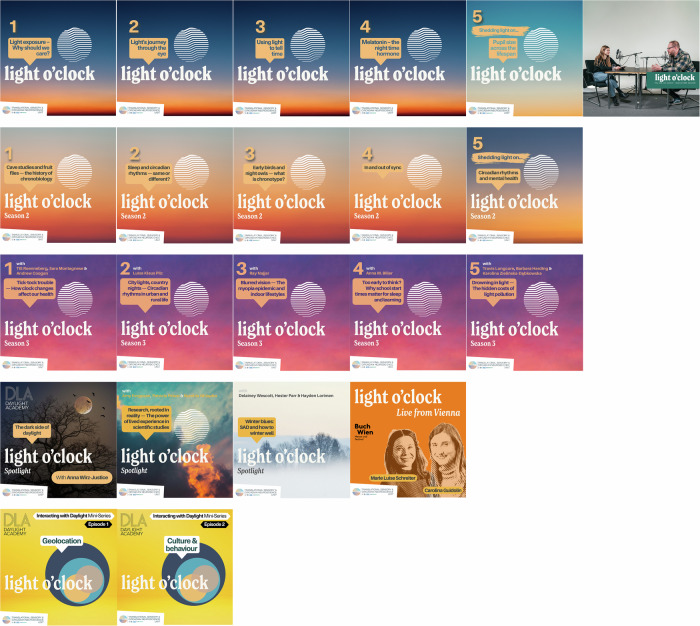

*Light O’Clock* was launched in 2024 as a science communication initiative in our research group (Table 2). The podcast was designed to make circadian science accessible to a wide audience by combining expert interviews, narrative storytelling and clear explanations of fundamental concepts. Each episode explores a specific theme related to light and biological rhythms—from melatonin and pupil size to circadian rhythms in mental health and the effects of light pollution—through conversations with leading researchers. The series aims to humanise science by showcasing the people behind the research, the questions that drive their work and the practical implications of their discoveries for everyday life.

Beyond its educational role, *Light O’Clock* serves as an experimental platform for innovative science communication. Episodes are released in themed seasons and special “Spotlight” editions that align with international events such as Daylight Awareness Week, enabling the podcast to respond dynamically to ongoing conversations in science and policy. The project also contributes to open research practice: all episodes are archived with a persistent infrastructure, ensuring long-term accessibility and citability, thereby recognising them as a research output.

## Podcasts as a tool to share emerging research with fellow scientists

Circadian science is a multidisciplinary field that encompasses molecular, clinical, behavioural and interventional dimensions of biological rhythms across a wide range of organisms, including plants, animals, and humans. While chronobiologists may be familiar with general biological rhythms topics by exposure at scientific meetings, their in-depth expertise typically lies within one specific subfield^10^. Keeping up with the literature of one’s own field is challenging; hence, consuming literature that is only loosely related to one’s own work is not a priority for researchers. This dynamic fosters research silos, limiting opportunities for cross-fertilisation of ideas^10,11^ and likely preventing collaborations that could yield important advances. In this context, podcasting offers an opportunity to bridge these divides through an auditory medium that is both accessible and refreshing in a research culture heavily reliant on screen-based reading. Conversational podcasts, in which a host interviews the author(s) of a recent study, can provide an accessible and engaging medium for delving into how the experiments were performed, the challenges encountered, and other details of research studies that are typically not captured in journal papers. For listeners (fellow chronobiologists), this can feel less like reading a paper and more like being part of an informal yet substantive exchange with the researchers themselves. Such exposure may prompt scientists to reassess their own work, spark new ideas, and inspire collaborations across domains. As such, podcasts created for the circadian and sleep community can deliver both convenient access to cutting-edge research and a sense of connection, strengthening the field’s collective capacity to innovate. Beyond the academic sphere, podcasts can also increase the visibility of chronobiological research to industry partners developing applications such as wearable devices or circadian-friendly lighting, fostering knowledge transfer and potential collaborations.

## Podcasts as a tool to communicate established findings and uncertainties to the public

Engaging the public with circadian and sleep science is key, given its direct societal relevance^10^. Circadian rhythms influence everyone’s daily life, and understanding the biological clock, how it is shaped by light exposure and how it affects alertness, sleep preferences, and overall health can empower individuals to make informed behavioural choices. Public awareness of circadian science thus has the potential to enhance health and well-being. Podcasting offers a uniquely accessible channel for this engagement. By fostering open dialogue with non-specialist audiences, podcasts can openly address both the knowns and unknowns of the field, humanise researchers, and demystify the scientific process^11^. Several formats lend themselves particularly well to this task:

- Thematic podcast series: Structured episodes that first introduce core principles of circadian biology (e.g., the existence of an internal clock, the role of light and other zeitgebers, and communication between central and peripheral clocks) and then build toward applied topics (e.g., social jet lag, night-shift work, and school start times). This approach equips audiences with a conceptual foundation, enabling them to understand the scientific rationale behind societal and policy decisions.
- Myth-debunking podcasts: These podcasts are designed to dismantle misconceptions and present the “knowns” and “unknowns” of circadian and sleep science. While media coverage often promotes circadian-friendly habits, information can be oversimplified or polarised (e.g., “blue light is bad”). Furthermore, persistent stereotypes, such as the notion that late chronotypes are lazier compared to earlier chronotypes, continue to shape perceptions^12^. Dispelling such myths can not only improve self-perception for later chronotypes but also encourage workplace policies, such as flexible hours. In this context, the “truth sandwich” method can be particularly successful^13^: a phenomenon of interest is explained (e.g., different chronotypes exist and are a result of our internal biological clock), followed by the myth to be debunked (later chronotypes are lazy and not favourable), and the phenomenon of interest is re-stated at the end of the episode (e.g., different chronotypes exist and are a result of our internal biological clock)^14^. Given the numerous open questions in circadian and sleep, these podcast formats must also be mindful of communicating the boundaries of current knowledge, presenting converging evidence alongside acknowledged gaps.
- Podcasts aimed at sharing the “knowledge of science”: Podcasting can also serve to humanise both the people behind the science and the research process itself, shifting the focus from simply transmitting *scientific knowledge* to sharing the *knowledge of science*—that is, how science is done in practice^11,15^. Increasing scientific literacy is not only about explaining key concepts (e.g., how circadian rhythms work) but also about making the realities of research visible: how circadian rhythms are measured, the time required for such measurements, and the inherent limitations of different approaches. This transparency can build public trust, dispel misconceptions about how science “magically” produces answers, and highlight the creativity, collaboration, and persistence involved in research. A podcast format well-suited to this aim is the researcher profile or “day in the life” interview, in which hosts discuss a scientist’s career path, daily work, and the behind-the-scenes challenges and triumphs of their research. By blending personal narrative with scientific discussion, this approach can make circadian and sleep scientists more relatable to the public.

## Podcasts as tools to support the incorporation of chronobiology into university curricula

Chronobiology is rarely included in standard life sciences or medical degree programs, and there is currently no consensus on which curricula should cover its key concepts^10^. While formal guidelines for chronobiology training should be integrated into relevant degrees such as Biology and Medicine, podcasts could serve as a practical interim solution. Specifically, we envision an open-access platform hosting chronobiology podcasts created by field experts, freely available for lecturers at different universities to incorporate into their teaching. This approach would complement existing video-based initiatives (see refs. ^12,16,17^) while providing an audio-only option, which can be more accessible for students with visual impairments. Lecturers could select episodes most relevant to their course, whether focused on molecular mechanisms, behavioural research, or applied aspects of the field. To meet students where they already spend substantial time, the platform could also integrate a social media strategy, where key segments from the podcasts are repurposed into a shorter form (e.g., “Shorts” or “Reels”) that highlights core concepts and directs students to the full episodes. This would also leverage podcasts as teaching tools in higher education, an approach shown to enhance learning outcomes, expand discipline-specific vocabulary, support revision, and spark student curiosity^18^. Furthermore, because students can listen to podcasts in parallel to performing other activities, they are perceived as less cognitively demanding compared to a traditional textbook^19^. As such, integrating podcasts into teaching resources could not only broaden chronobiology’s presence in university curricula but also engage students in a flexible, accessible, and appealing format.

## Podcasts as tools to promote behaviour change as part of digital behavioural interventions

One of the goals of circadian and sleep scientists is not only to share knowledge with the public but also to encourage behaviours that support healthy circadian function, such as maximising morning light exposure and reducing evening light exposure. Podcasts offer a dynamic medium for achieving both aims: they can deliver health information in an engaging format while simultaneously prompting behaviour change. A recent scoping review of 38 studies investigating podcasts as tools for promoting health-related behaviours found preliminary evidence for their effectiveness in areas such as physical activity and dietary habits^20^. Beyond potentially influencing behaviour through exposure to health messaging, podcasts could also function as scalable recruitment and engagement tools to enable large-scale testing of behavioural interventions. A notable related example comes from the podcast series *Body Electric*, produced by National Public Radio, which explores the effects of technology use and modern sedentary lifestyles on our health. The podcast producers collaborated with researchers at Columbia University Medical Centre who had found that, in the laboratory, regular movement breaks can counteract the harmful effects of daytime sitting. This collaboration enabled the recruitment of a large sample for a follow-up study to test whether these benefits translate to real-world conditions: >20,000 podcast listeners enroled in the study^21^ and reported their experience participating in the podcast. In the context of circadian and sleep science, similar strategies could be applied to promote evidence-based habits and/or test targeted interventions, particularly when integrated with other digital tools such as smartphone applications. For example, on the one hand, existing podcasts on circadian and sleep science could collaborate with research institutions and encourage listeners to participate in ongoing real-world clinical trials related to sleep and circadian science. On the other hand, podcasts could also be incorporated into existing app-based interventions designed to improve circadian and sleep health in populations at elevated risk of circadian disruption, such as shift workers or university students, as a strategy to build awareness and communicate the science behind a given intervention.

## Challenges and potential pitfalls of science podcasting

Below, we highlight some challenges and potential pitfalls of podcasting as a tool for science communication and specifically for health-related topics. A detailed overview of challenges faced by academic podcasting in general is beyond the scoping of this article and can be found elsewhere^11^. A central issue is the evaluation of podcast quality and credibility. Because podcast production has a relatively low barrier to entry, content can vary widely in accuracy, balance, and evidentiary support, raising concerns about the credibility and objectivity of claims made by hosts and guests. This is particularly salient for health- and medical-related topics, where misleading or weakly supported advice is common in adjacent digital media environments^22^. In addition, many health-related podcasts are supported by commercial sponsorship, which may introduce conflicts of interest and subtly shape the framing of advice, even when sponsorship is disclosed.

To support more systematic appraisal, Nelson and Faux (2016)^23^ proposed the *Podcast Evaluation Rubric* that considers five dimensions: (1) expertise and credentials, (2) accuracy, (3) quality of information, (4) production quality, and (5) currency of information. This framework provides a useful starting point for assessing individual episodes, but important questions remain about implementation and reach: who applies these criteria at scale, and how are the results communicated to audiences in an accessible way? The general public, who is the primary audience for science podcasts^1^, may not have the time or specialist knowledge required to evaluate claims. Conversely, although individual scientists can correct misinformation, their efforts may not reach everyone exposed to problematic content. While organised fact-checking initiatives for science news exist (e.g., *Science Feedback and AuthentiSci*), the extent to which comparable processes are applied to podcasts is unclear. Developing approaches to transparently signal credibility by including episode-level assessments could therefore be a valuable step toward reducing misinformation in this rapidly growing medium.

A second challenge, especially for health-related science podcasts, concerns audience reach: are podcasts being heard by those who would benefit most from the information? While an analysis of 952 science podcasts produced between 2004 and 2018 reported that most (77%) targeted the “general public”^1^, very little is known about the demographics or psychographics of science podcast listeners. A recent study^24^ examined listener demographics (*n* = 226) and found that most participants (62.8%) were female; the predominant age group was 25–34 years; and the most commonly reported highest level of education was a Bachelor’s degree (34.9%). It is therefore plausible that science podcast audiences are, on average, relatively well educated and disproportionately health-oriented, or already motivated to seek behaviour-relevant information, which may limit reach into groups with lower awareness or fewer resources. In the case of circadian science podcasts, for example, a shift worker who already notices sleep disruption and health impacts may be more likely to seek out a circadian science podcast than a colleague who has not connected shift schedules with well-being, despite the latter potentially benefiting substantially from the same information. Further studies examining the motivations for, and determinants of, engagement with science podcasts could help identify strategies to reach audiences beyond those already motivated or health-oriented.

## Conclusion

Podcasting offers a versatile and accessible medium for advancing chronobiology communication between different stakeholders. By adopting formats such as conversational interviews, thematic series, myth-debunking episodes, researcher profiles, and campaign-driven interventions, chronobiologists can extend the reach, depth, and societal impact of their work.

The formats discussed here are by no means exhaustive, and the field of academic podcasting is still evolving. We encourage chronobiologists to be creative, audience-focused and set aside the fear of “doing it right”, as authenticity often matters more than strict adherence to a preconceived science communication template. Chronobiologists and communication professionals should explore podcasting not merely as an outreach tool but as part of a broader strategy for integrating research, education and public engagement.

At the same time, it is important to recognise that academic podcasting comes with both opportunities and challenges^11^. These include questions around quality control, discoverability, sustainability, and, critically, funding. Producing high-quality podcasts requires time, skills, and resources, yet dedicated funding streams for academic podcasting remain scarce. Addressing these barriers will be essential if podcasts are to become a sustainable and impactful part of science communication strategies. Developing shared guidelines and evaluation frameworks for academic podcasts could help establish their role as legitimate research communication outputs.

## Data Availability

No datasets were generated or analysed during the current study.
